# Evaluation of a standardised Vi poly-l-lysine ELISA for serology of Vi capsular polysaccharide antibodies

**DOI:** 10.1016/j.biologicals.2020.05.002

**Published:** 2020-07

**Authors:** Peter Rigsby, Emma Beamish, Jason Hockley, Eleanor Atkinson, Krisztina Hitri, Elizabeth Jones, Jae Seung Yang, Firdausi Qadri, Novilia S. Bachtiar, Sean C. Elias, Akshay Goel, Ravipratapnarayan Mishra, Raju Dugyala, Marcela F. Pasetti, James E. Meiring, Maurice Mbewe, Melita A. Gordon, Andrew J. Pollard, Alastair Logan, Sjoerd Rijpkema, the anti-Vi IgG working group

**Affiliations:** a Biostatistics Group, National Institute for Biological Standards and Control, Potters Bar, Hertfordshire, EN6 3QG, UK; b Division of Bacteriology, National Institute for Biological Standards and Control, Potters Bar, Hertfordshire, EN6 3QG, UK; c Oxford Vaccine Group, Department of Paediatrics, University of Oxford and the NIHR Oxford Biomedical Research Centre, Oxford, OX3 7LE, United Kingdom; d Clinical Immunology, International Vaccine Institute, SNU Research Park, 1 Gwanak-Ro, Gwanak-Gu, Seoul, Republic of Korea; e International Centre for Diarrhoeal Disease Research Bangladesh, 68 Shaheed Tajuddin Ahmed Sharani, Mohakhali, Dhaka, 1212, Bangladesh; f Clinical Trial Department, Surveillance & Clinical Trial Division, Bio Farma, Jl.Pasteur No.28, Bandung, Indonesia; g Jenner Institute, Nuffield Department of Medicine, University of Oxford, Old Road Campus Research Building, Roosevelt Drive, Oxford, OX3 7DQ, United Kingdom; h R&D, Biological E. Ltd, MN Park, Genome Valley, Shameerpet, Hyderabad, 500078, Telangana, India; i Quality Operations, Bharat Biotech International Ltd, Genome Valley, Shameerpet, Hyderabad, 500078, Telangana, India; j Center for Vaccine Development, University of Maryland Baltimore, 685 West Baltimore Street, Room 480, Baltimore, MD, USA; k Malawi-Liverpool Wellcome Trust Clinical Research Programme, College of Medicine, Blantyre, Malawi

**Keywords:** ELISA, IgG, Typhoid, Poly-l-lysine, Polysaccharide, Vi, AD, Assay Diluent, BB, Blocking Buffer, CV, Coefficient of Variation, ECBS, Expert Committee on Biological Standardization, GCV, Geometric Coefficient of Variation, GM, Geometric Mean, IU, International Unit, IS, International Standard, PLL, poly-l-lysine, Vi, capsular Vi polysaccharide

## Abstract

Typhoid vaccines based on protein-conjugated capsular Vi polysaccharide (TCVs) prevent typhoid in infants and young children. Analysis of the serum anti-Vi IgG response following immunisation against typhoid confirms the immunogenicity of TCVs and forms an important part of the pathway to licensing. Comparative studies could expedite the licencing process, and the availability of a standardised ELISA method alongside the 1st International Standard (IS) 16/138 for anti-typhoid capsular Vi polysaccharide IgG (human) will facilitate this process. To this end, a non-commercial ELISA based on a coat of Vi and poly-l-lysine (Vi-PLL ELISA) was evaluated by 10 laboratories. Eight serum samples, including IS 16/138, were tested in the standardised Vi-PLL ELISA (*n* = 10), a commercial Vi ELISA (*n* = 3) and a biotinylated Vi ELISA (*n* = 1). Valid estimates of potencies relative to IS 16/138 were obtained for all samples in the Vi-PLL ELISA and the commercial ELISA, with good repeatability and reproducibility evident from the study results and concordant estimates obtained by the two ELISA methods. The study demonstrates that the Vi-PLL ELISA can be used in clinical trial studies to determine the immunogenicity of TCVs.

## 1 Introduction

Typhoid fever is caused by an infection with *Salmonella enterica* subspecies *enterica* serovar Typhi (*S. typhi*). In developing countries, typhoid disease particularly affects children and infants and is a considerable cause of morbidity and mortality. A recent study estimated a global incidence of 12 million cases of typhoid and 129,000 deaths each year [1].

Capsular Vi polysaccharide (Vi) is a virulence factor of *S. typhi* and considered a protective antigen in typhoid vaccines [2,3]. Vaccination is the most cost-effective strategy to prevent and control typhoid fever in endemic areas and in areas where anti-microbial resistant strains reside. In the 1980s, oral live attenuated Ty21a and the injectable plain Vi vaccines were licensed and have been on the market since. Unfortunately, these vaccines suffer two drawbacks: they are not suitable for use in children under five and two years of age respectively, and plain Vi vaccines are unable to induce a booster response in adults and children, and for those at risk (e.g. lab workers) a repeat immunisation is required every 3 years [[4], [5], [6]]. Conjugation of Vi to a carrier protein such as a bacterial toxoid remedied these shortcomings: a prototype typhoid conjugate vaccine (TCV) consisting of Vi conjugated to recombinant *Pseudomonas aeruginosa* exoprotein A was proven to be immunogenic and induced a booster response in young children, was safe and efficacious in pre-school age children and infants and induced a long lasting anti-Vi IgG response [[7], [8], [9]]. The success of this TCV prompted the World Health Organization (WHO) to draft the WHO guideline on the quality, safety and efficacy of TCVs, which provides a framework to evaluate TCVs, compare clinical trial studies and analyse the safety, consistency and potency of TCVs by physicochemical assays and immunoassays [10,11].

Three TCVs consisting of Vi conjugated to tetanus toxoid (Vi-TT) are licensed in India on the bases of immunogenicity and safety studies [[12], [13], [14]]. Indeed, the presence of anti-Vi IgG following immunisation with a Vi vaccine is considered a correlate of protection [[7], [8], [9],^11^,^15^]. Studies in a controlled human infection model (CHIM), showed the Vi-TT vaccine to have similar effectiveness compared with plain Vi vaccine, but with improved immunological properties [16]. Currently, three TCVs are progressing through clinical trials, three are approaching licensure, and a further five are at the pre-clinical stage [14]. Recently, a phase III field trial in Nepal of Vi-TT showed it is efficacious and reduces the incidence of *S. typhi* in children [17], and, in Hyderabad (Pakistan) a mass immunisation campaign with Vi-TT is undertaken to control an outbreak of antimicrobial resistant variants [18].

To expedite the licensing process for new TCVs, we produced and evaluated the first International Standard (IS) for anti-Vi IgG (human), 16/138 [19]. A collaborative study showed that performance of IS 16/138, US reference reagent Vi-IgG_R1, 2011_ and individual post-immunisation sera was most consistent in the commercial VaccZyme Human Anti-*Salmonella typhi* Vi IgG ELISA (Binding Site, UK) followed by the Vi ELISA pre-coated with poly-l-lysine (Vi-PLL). The poor performance of ELISAs based on a coat of Vi only was noted in this study, its predecessor as well as an earlier study [[19], [20], [21]]. The latter observation agrees with previous studies that reported poor binding of bacterial polysaccharides to the micro-plate surface resulting in inconsistent ELISA results, which can be mitigated by pre- or co-coating the polysaccharide with a protein or by chemical modification of the polysaccharide [20,22,23]*.* Poly-l-lysine is a representative of nucleic acid binding polymers and has been used to bind DNA and RNA avidly on the basis of their charge and thus increase their adherence to the solid phase [24]. Like these polymers, polysaccharides have a negative charge and a PLL coating will capture these molecules efficiently. Poly-l-lysine was chosen as co-coating protein over human serum albumin, because the Vi PLL ELISA showed superior assay precision in a comparative study [19].

Following the establishment of IS 16/138, the WHO Expert Committee on Biological Standardization (ECBS) requested a study in order to establish whether a non-commercial Vi ELISA based on these principles could be a reliable and robust alternative to the commercial VaccZyme ELISA [25]. The current study was designed to evaluate the Vi-PLL ELISA as a generic and non-commercial alternative to the VaccZyme ELISA. The performance and reproducibility of the Vi-PLL ELISA alongside the commercial VaccZyme ELISA was assessed by 10 laboratories, using a set of pre- and post-immunisation sera from volunteers immunized with licenced Vi vaccines, as described previously, IS 16/138 and working standard 10/126 [19].

## 2 Materials and methods

### 2.1 Participating laboratories and assay codification

Ten participants, including vaccine manufacturers, national control laboratories and research laboratories from seven countries tested the study samples and supplied data for this study (see acknowledgments section for details). Throughout the study, laboratories were identified by a randomly assigned laboratory code number to maintain confidentiality. Data were collected and analysed at NIBSC. A study protocol and the procedure for the Vi-PLL ELISA was sent to each participant along with six suggested plate layouts to be used.

### 2.2 Samples used in the collaborative study

Each participant received a sample set comprising one ampoule of IS 16/138, coded ampoules (G to M), and one ampoule of 10/126. A brief characterisation of the study samples, study codes, NIBSC codes and their reactivity in the VaccZyme ELISA based on preliminary testing is given in Table 1. Samples IS 16/138, 10/126 and coded samples G, I and J had been used previously [19].

**Table 1:** Characterisation of samples used in this study.

Approximate activity in VaccZyme ELISA	NIBSC code	Description		
			Kit control^a^	IS 16/138
IS 16/138	NIBSC 16/138	Pooled anti-Vi IgG sera from 16 volunteers	631 EU mL^−1^^b^	100 IU mL^−1^^b^
10/126	NIBSC 10/126	Pooled anti-Vi IgG sera from 9 volunteers	842 EU mL^−1^^b^	109 IU mL^−1^^b^
G	NIBSC 16/150	Post-Typbar TCV vaccination serum	350 EU mL^−1^^b^	38 IU mL^−1^^b^
H	NIBSC 16/138	Pooled anti-Vi IgG sera from 16 volunteers	631 EU mL^−1^^b^	97 IU mL^−1^^b^
I	NIBSC 16/144	Post-Typbar TCV vaccination serum	345 EU mL^−1^^b^	25 IU mL^−1^^b^
J	NIBSC 16/146	Post-Typhim vaccination serum	524 EU mL^−1^^b^	62 IU mL^−1^^b^
K	NIBSC 16/148	Post-Typhim vaccination serum	150 EU mL^−1^^c^	–
L	NIBSC 16/168	Post-Typbar TCV vaccination serum	3876 EU mL^−1^^c^	–
M	NIBSC 16/180	Post-Typhim vaccination serum	61 EU mL^−1^^c^	–

a Supplied by manufacturer.

b Rijpkema et al. 2018.

c Preliminary estimate based on 2 VaccZyme ELISA runs performed at NIBSC.

Coded samples and IS 16/138 were produced using sera donated by volunteers immunized with WHO prequalified and licensed vaccines either a plain Vi vaccine or a Vi-TT conjugate vaccine, who were recruited by the Oxford Vaccine Group (OVG) [16,19]. Sera used for 10/126, originated from clinical trial volunteers immunized with the same Vi-TT conjugate vaccine and sera were donated by Bharat Biotech Limited International of India [19,20].

All samples tested negative for antibodies to HIV 1/2 and hepatitis C and hepatitis B surface antigen. Freeze dried samples IS 16/138, 10/126 and G to M were reconstituted as described in the instructions for use.

To allow for a standardised Vi-PLL ELISA to be performed by participants, each of them received essential reagents which met the designated specifications of the assay: one ampoule of IS 12/244 for Vi from *Citrobacter freundii*, one ampoule of mouse monoclonal anti-human IgG Fc alkaline phosphatase (AP) conjugate, one vial of poly-l-lysine and one vial of 4-nitrophenyl phosphate disodium salt hexahydrate substrate. In one instance, BRIJ-35 was supplied as well. VaccZyme ELISA kits were sent to 3 participants. All samples and reagents were sent on dry ice or 2–8 °C as prescribed.

### 2.3 ELISAs

All participants tested the study samples in the Vi-PLL ELISA. In addition, three participants used the VaccZyme ELISA and one participant carried out an in-house biotinylated Vi ELISA. The code and a brief description of the ELISAs are given in Table 2. ELISA procedures are described below. Six different plate layouts were provided, and samples were tested over two days with 3 plates on each day. Each participant was asked to prepare starting dilutions of samples at 1:100 in appropriate assay diluents, except for IS 16/138, which had a starting dilution of 1:25 and sample L which had a starting dilution of 1:400.

**Table 2:** Format of Vi ELISAs used by participants.

Laboratory code	ELISA method		Vi characteristics			Antigen coating procedure
	Name	Format		Status	Biological origin	
1,5,6,	VaccZyme	Indirect		Not disclosed	*S.* Typhi	Procedure not disclosed
1,2,3,4,5,6,7,8,9,10	Vi poly-l-lysine	Indirect		Native	*C. freundii*	Pre-coat with poly–l-Lysine to bind Vi
6	Biotinylated Vi	Capture		Biotinylated	*S.* Typhi	Streptavidin coat to bind biotinylated Vi

#### 2.3.1 VaccZyme ELISA

The VaccZyme ELISA was carried out according to the manufacturer's instructions (Binding Site). The VaccZyme ELISA is based on Vi of *S.* Typhi. Five calibrators, covering a range from 7.4, 22.2, 66.7, 200 and 600 EU mL^−1^, and a high and a low positive control were included in each run. The assay has a sensitivity of 7.4 EU mL^−1^. Plates were read at OD_450nm_.

#### 2.3.2 Vi poly-l-lysine ELISA

The procedure for the Vi-PLL ELISA is a modification of the assay described by Szu et al. [26] by the addition of a pre-coating step with high molecular weight poly-l-lysine hydrobromide (P1399, Sigma) [24,27], followed by a coat of Vi from *C. freundii* (NIBSC 12/244). In brief, 100 μL of 10 μg poly-l-lysine mL^−1^ in PBS was added to wells of a Nunc Maxisorp plate, incubated at RT for 2 h and washed 6x with 0.85% NaCl and 0.1% Brij35 in ultrapure water (NaCl-Brij 35), 100 μL of 2 μg Vi mL^−1^ in PBS was added to the wells. Plates were incubated overnight at 37 °C and washed 6x with NaCl-Brij 35. 250 μL 1% BSA in PBS (Blocking Buffer, BB) was added to each well. Plates were incubated for 1 h at 37 °C and washed six times. Dilutions of test samples were prepared in assay diluent 1 (AD1: BB with 0.1% Brij35). 100 μL of diluted tested sample was dispensed to appropriate wells of the plate. Samples were incubated at 37 °C for 1 h, and washed 6x with NaCl-Brij 35. Then 100 μL of mouse monoclonal Anti-Human IgG Fc AP conjugate diluted 1:5000 in AD1 was added to each well, incubated at 37 °C for 1 h and washed 6x with NaCl-Brij 35. 100 μL 4-Nitrophenyl Phosphate disodium salt hexahydrate substrate (N 7653, Sigma) in Tris/3 mM Mg-buffer (pH 9.8) was added per well and incubated at RT in dark for 1 h, followed by the addition of 50 μL 3 M NaOH stop solution to each well. The plate was read at OD_405nm_.

A post-study questionnaire revealed that participants adhered closely to the procedure described above (results not shown). However, some variations occurred: plates were rinsed either by hand or by plate washer, and if a plate washer is used a 180° turn of the plate after three rinses should be carried out. Laboratories 1 and 10 sealed ELISA plates during incubation steps but most laboratories covered plates with lids.

#### 2.3.3 Biotinylated Vi ELISA

The procedure carried out by laboratory 6 is a minor modification of the ELISA described by Ferry et al. [28]. The plate surface is coated with streptavidin to capture biotinylated Vi from *S. typhi* (Fina Biosolutions LLC). In brief, streptavidin from *Streptomyces avidinii* (Sigma, S4762) was prepared in a solution of 3 μg mL^−1^ and 100 μL was added to each well of flat bottom microtiter plates (Nunc Maxisorp) and incubated uncovered at 37 °C overnight, allowing the solution to evaporate to dryness. Pre-coated plates were stored at 4 °C until used for coating. For antigen coating, 2 μg mL^−1^ of biotinylated *S*. Typhi Vi PS produced locally was prepared in PBS (pH 7.4) and 100 μL was added to the wells of pre-treated plates. Plates were incubated for 3 h at 37 °C and washed with PBS-T and wells were blocked overnight with 250 μL PBS containing 10% non-fat dry milk at 4 °C. Plates were washed 6x with PBS-T with 2 min soaking period in between washes. Sera and reference reagents were diluted in AD2 (10% non-fat dry milk (w/v) in PBS (pH 7.3–7.5) and 0.05% Tween-20) and added to the plates. All plates were incubated for 1 h at 37 °C. Following incubation and washing as described above, plates were incubated with HRP-labelled goat anti-Human IgG (Jackson Immunoresearch, 109-035-008) diluted in AD2 for 1 h at 37 °C. The plates were washed 6x with PBS-T, and 100 μl of TMB Microwell Peroxidase Substrate (KPL, SeraCare Life Sciences Inc) was added to each well, incubating for 15 min in the dark (with agitation). The reaction was stopped by adding 100 μL of 1 M Phosphoric acid per well. Plates were read immediately at OD_450nm_.

### 2.4 Statistical analysis

**Fig. 1:**
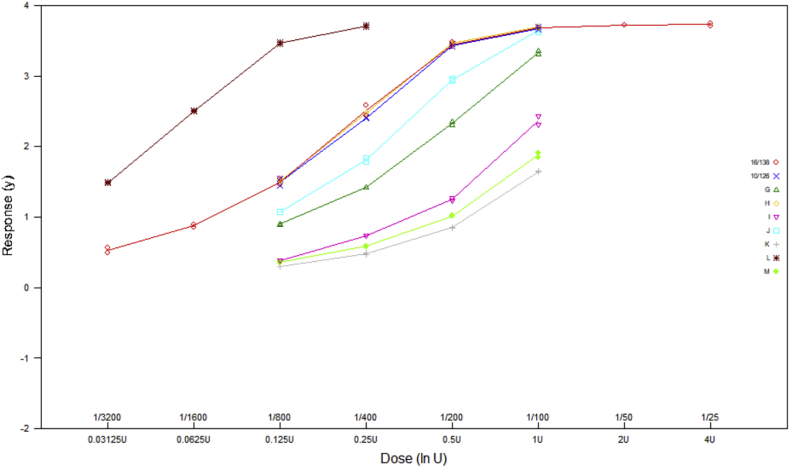
Example of data reported for single Vi-PLL ELISA plate.

Raw data from all individual plates were analysed with a four-parameter logistic model (sigmoid curves) in order to determine the potencies of study samples relative to IS 16/138. An example of the data obtained from a single plate is shown in Fig. 1. Analysis was performed using EDQM CombiStats Software Version 5.0 [29]. Acceptable parallelism of dose-response relationships for a test sample and IS 16/138 was concluded if the slope ratio calculated by CombiStats was within the range [0.80, 1.25]. Acceptable precision was concluded for each relative potency estimate if it had a 95% confidence interval no wider than 80–125% of the estimate. In addition to these criteria for individual samples, each plate was only considered acceptable if valid potency estimates were obtained for sample H (coded duplicate of IS 16/138) and 10/126, both within 66.7–150% of their assigned values of 100 IU mL^−1^ and 109 IU mL^−1^ respectively. No potency estimates have been reported where samples or plates did not meet these criteria. It should be noted that the validity criteria were intended for use in the analysis of data from this study only, in order to apply consistent criteria to all laboratories and assess their relative performance. They should not be interpreted as suitable for routine use in the assessment of assay validity within the collaborating laboratories unless suitably justified.

Data from laboratory 3 indicated IS 16/138 was not tested at the appropriate requested dilutions, therefore potencies of study samples relative to sample H, the coded duplicate of IS 16/138, were analysed using a parallel line model. The same assay validity criteria as described above were applied.

All mean results shown in this report are unweighted geometric means (GM). Variability has been expressed using geometric coefficients of variation (GCV = {10^s^-1} × 100%, where s is the standard deviation of the log_10_ transformed estimates). Individual assay estimates of relative potency were log transformed and a mixed effects model used to determine intra-laboratory and inter-laboratory variance components (also expressed as % GCV) for each sample.

Further assessment of agreement in geometric mean results for each pair of laboratories was performed by calculating Lin's concordance correlation coefficient [30,31] with log transformed data. Calculations for this were performed using the R package ‘DescTools’ [32]. A value of 1 for this coefficient indicates perfect agreement between the two laboratories.

## 3 Results

### 3.1 Performance of individual laboratories

**Fig. 2:**
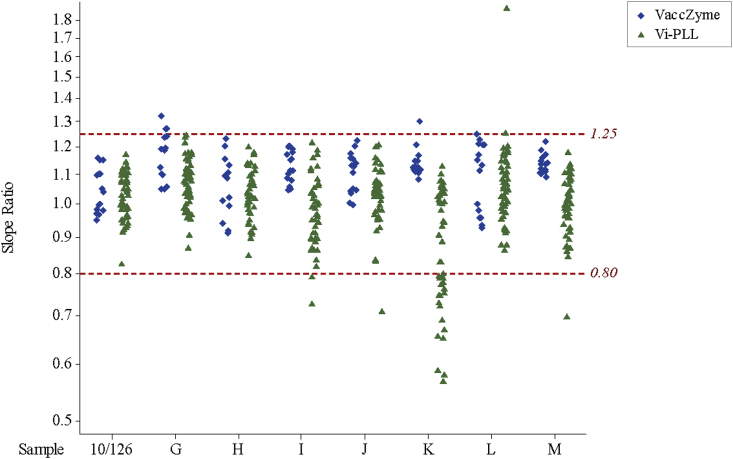
Overview of slope ratios of samples 10/126 and G-M relative to IS 16/138 when tested in the Vi-PLL ELISA and Vacczyme ELISA. Sample H is a coded duplicate of 16/138.

The number of valid relative potency estimates obtained for each sample in each laboratory are shown in Table 3 and cases where fewer than three valid estimates were obtained are highlighted. The intra-laboratory (between-plate) variability for each sample is shown in Table 4 and cases where the GCV value exceeds 25% are highlighted. Details of all individual assay results and outcomes are provided in the supplementary table (attached separately). A summary of the causes of assay invalidity when applying the criteria described above is given in Table 5. A large number of the invalid assays (21 out of 53) were due to the non-parallelism of sample K and IS 16/138 in Vi-PLL ELISAs, particularly those performed by laboratories 2, 3, 5 and 7, with slope ratios for sample K falling below the acceptable lower limit of 0.80 (see Fig. 2). This phenomenon appears to be unique for sample K and was not observed for sample M which has a similar low relative potency. Apart from this observation regarding sample K, the reasons for assay invalidity are mixed (e.g. non-parallelism, 10/126 giving result higher or lower than expected range) and related to the performance quality of the laboratory.

**Table 3:** Number of valid potency estimates relative to IS 16/138 obtained out of 6 runs for each study sample in the VaccZyme, Vi poly-l-lysine and biotinylated-Vi ELISAs.

ELISA	Laboratory code	Valid potency estimates per study sample							
		10/126	G	H^a^	I	J	K	L	M
Vi poly-l-lysine	1	6	6	6	6	6	6	6	6
	2	6	6	6	4	6	3	6	5
	3	6	6	nt^b^	6	6	1	6	5
	4	3	3	3	3	2	3	3	2
	5	6	5	6	5	6	1	6	6
	6	3	3	3	3	3	3	3	3
	7	6	6	6	5	5	0	5	6
	8	5	5	5	5	5	5	5	5
	9	5	5	5	4	4	3	4	4
	10	3	3	3	3	3	2	3	3
VaccZyme	1	6	3	6	6	6	6	5	6
	5	3	3	3	3	3	3	3	3
	6	6	6	6	6	6	5	6	6
Biotinylated-Vi	6	6	6	6	6	6	5	6	6

a ^:^ Coded duplicate of IS 16/138.

b ^:^ not tested.

**Table 4:** Intra-laboratory variation for potency estimates of study samples in the VaccZyme, Vi poly-l-lysine and biotinylated-Vi ELISAs.

ELISA	Laboratory code	Intra-laboratory variation for study samples as % GCV^a^							
		10/126	G	H^b^	I	J	K	L	M
Vi poly-l-lysine	1	5	6	6	9	7	9	6	9
	2	18	15	12	6	20	22	16	23
	3	6	10	nt^c^	12	8	nc^d^	8	7
	4	22	27	9	12	nc^d^	42	44	nc^d^
	5	12	27	10	11	16	nc^d^	28	10
	6	9	8	11	21	9	15	25	2
	7	12	25	22	51	29	nc^d^	47	22
	8	5	10	12	3	6	5	8	6
	9	17	12	12	10	14	10	13	13
	10	7	8	10	16	11	nc^d^	17	2
VaccZyme	1	10	9	8	10	8	7	8	6
	5	18	14	2	5	25	59	41	32
	6	5	6	3	5	2	2	5	5
Biotinylated-Vi	6	5	5	2	7	8	2	4	4

a Geometric Coefficient of Variation.

b Coded duplicate of IS 16/138.

c Not tested.

d Not calculated as fewer than 3 valid potency estimates were obtained for the sample.

**Table 5:** Causes of assay invalidity in Vi-PLL ELISA.

Invalidity reason	Number of invalid cases
Non-parallel (slope ratio < 0.80)	26^a^
Non-parallel (slope ratio > 1.25)	**7**
10/126 low (<72.7 IU mL^−1^)	2
10/126 high (>163.5 IU mL^−1^)	4
10/126 non-parallel (slope ratio < 0.80)	0
10/126 non-parallel (slope ratio > 1.25)	0
Sample H low (<66.7 IU mL^−1^)	0
Sample H high (>150 IU mL^−1^)	4
H non-parallel (slope ratio < 0.80)	1
H non-parallel (slope ratio > 1.25)	2
No convergence (cannot fit model)	4
Poor plate precision (wide CI)	3
Total of invalid cases	53 (out of 474^b^)

a 21 out of 26 cases are for sample K in the Vi-PLL-ELISA by labs 2, 3, 5 and 7.

b Maximum number of potency estimates possible in this study.

If these criteria are used to define a good assay performance (i.e. three or more valid estimates for all samples and GCV values ≤ 25%), the Vi-PLL ELISA of laboratories 1, 2, 6, 8, 9, the VaccZyme ELISA of laboratories 1 and 6, and the biotinylated Vi ELISA of laboratory 6 were all considered to have been performed to a good standard.

Laboratories 3, 5 and 10 also appeared to perform the Vi-PLL ELISA to an acceptable standard, obtaining three or more valid estimates for all samples except for K. All GCV values for these laboratories were also ≤25%, with the exception of samples G and L by laboratory 5 (GCV values of 27% and 28% respectively).

Several high GCV values (>40%) or a low number of valid estimates were obtained by laboratories 4 and 7 for the Vi-PLL ELISA and by laboratory 5 for the VaccZyme ELISA, therefore the quality of the performances of these laboratories was considered doubtful for these assays. Based on our assessment, the data from these laboratories were excluded from further calculations to assess inter-laboratory variability (reproducibility) and the comparison of the two ELISAs.

### 3.2 Comparison of Vi poly-l-lysine and VaccZyme ELISAs

**Fig. 3:**
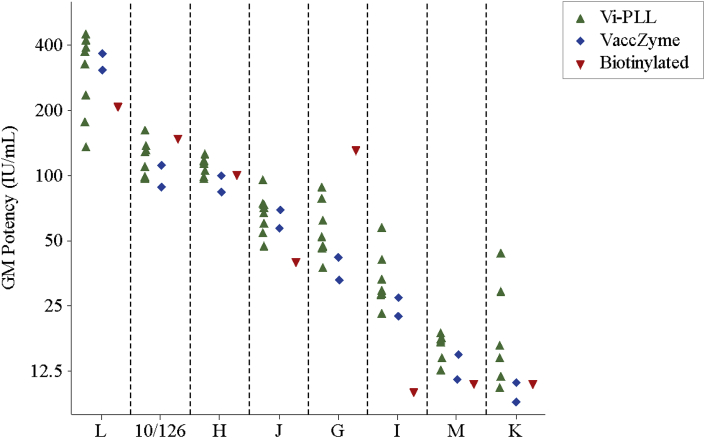
Geometric mean potency estimates for study samples relative to IS 16/138 in IU mL^−1^ for the VaccZyme ELISA performed by laboratories 1 and 6, the Vi poly-l-lysine ELISA (Vi-PLL) performed by laboratories 1, 3, 5, 6, 8, 9 and 10, and biotinylated-Vi ELISA performed by laboratory 6.

Table 6 and Fig. 3 show the GM potency estimates in IU mL^−1^ for each of the study samples as reported by each laboratory. Table 7 shows the concordance correlation coefficients for each laboratory pair, with values in excess 0.80, 0.90 and 0.95 shaded to indicate increasing levels of concordance in results obtained across the panel of samples tested in the Vi-PLL and VaccZyme ELISAs. The majority of values for laboratory 2 were <0.80, showing poor concordance with other laboratories. Excellent concordance was observed between laboratories 1, 3 and 8 performing the Vi-PLL ELISA and laboratories 1 and 6 performing the VaccZyme ELISA (all values > 0.95). In addition, low intra-laboratory variability was observed for these laboratories, with average GCV values < 10% (see Table 4).

**Table 6:** Geometric mean potency estimates of study samples relative to IS 16/138 in the VaccZyme, Vi poly-l-lysine and biotinylated-Vi ELISAs.

ELISA	Laboratory code	GM^a^ potency estimates for study samples in IU mL^−1^							
		10/126	G	H^b^	I	J	K	L	M
Vi poly-l-lysine	1	95	46	98	29	70	10	372	12
	2	97	37	96	57	47	43	134	19
	3	108	47	nt^c^	34	68	nc^d^	329	15
	5	130	62	115	28	74	nc^d^	445	18
	6	131	80	112	21	50	10	175	14
	8	98	49	105	22	72	14	384	17
	9	126	47	125	28	99	32	407	17
	10	150	75	114	38	59	nc^d^	220	16
VaccZyme	1	89	33	84	22	57	9	307	11
	6	111	42	100	27	69	11	365	15
Biotinylated-Vi	6	149	131	101	10	40	11	208	11

a Geometric Mean.

b Coded duplicate of IS 16/138.

c Not tested.

d Not calculated as < 3 valid potency estimates were obtained for sample.

**Table 7:**
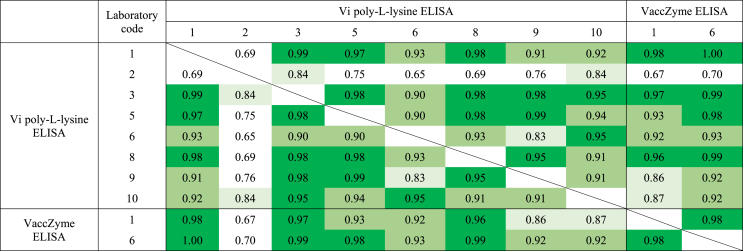
Concordance correlation coefficients in laboratory pairs for log potencies relative to IS 16/138 in the VaccZyme and Vi poly-l-lysine ELISAs.

**Fig. 4:**
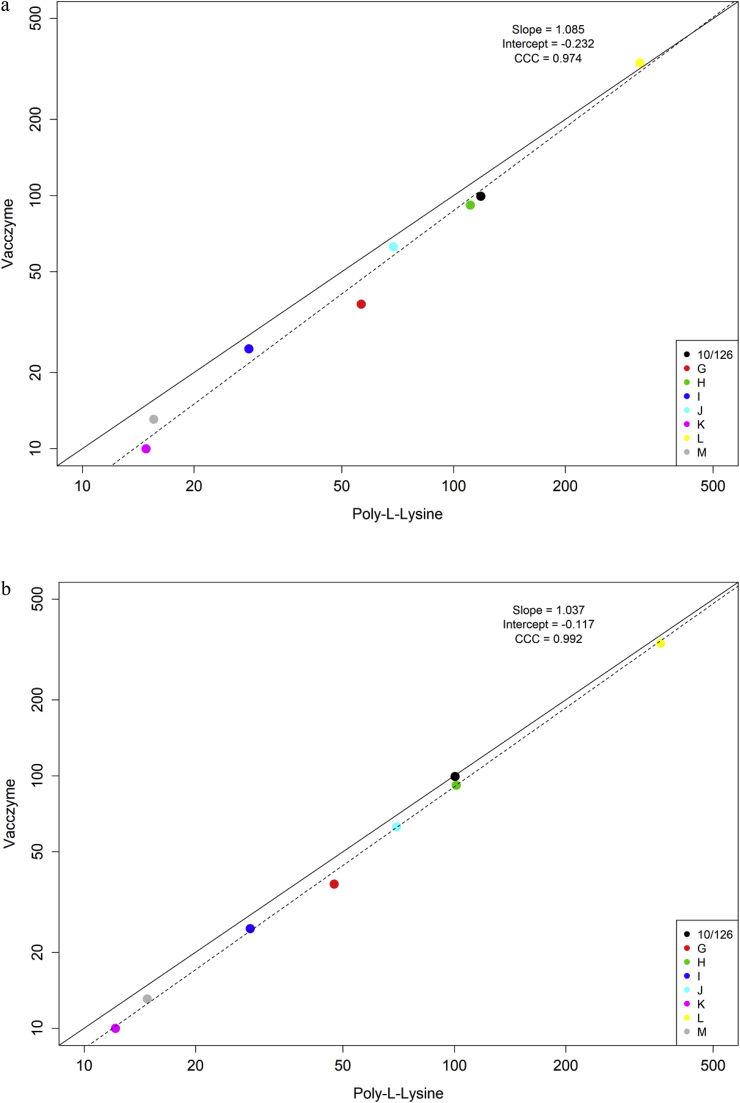
**a** Comparison of overall geometric mean potency estimates in IU mL^−1^ relative to IS 16/138 by the Vi poly-l-lysine ELISA performed by laboratories 1, 3, 5, 6, 8, 9 and 10 and the VaccZyme ELISA performed by laboratories 1 and 6. **4b**: Comparison of overall geometric mean potency estimates in IU mL^−1^ relative to IS 16/138 by the Vi poly-l-lysine ELISA performed by laboratories 1, 3 and 8 and the VaccZyme ELISA performed by laboratories 1 and 6.

A summary of overall GM potency estimates from each ELISA, excluding data from laboratory 2 for the Vi-PLL ELISA, is shown in Table 8 and Fig. 4a. An illustration of the highest levels of agreement and reproducibility that can be achieved for the Vi-PLL and VaccZyme ELISAs reached by laboratories which demonstrated a superior level of performance in this study is given in Table 8 and Fig. 4b. A high degree of concordance in geometric mean estimates was evident between the two ELISA methods (Lin's concordance correlation coefficient 0.974 and 0.992 in Fig. 4a and b, respectively).

**Table 8:** Summary of overall geometric mean potency estimates of study samples relative to IS 16/138 in the VaccZyme and Vi poly-l-lysine ELISAs.

Data From	Vi poly-l-lysine ELISA						VaccZyme ELISA		
	Laboratories 1, 3, 5, 6, 8, 9 and 10			Laboratories 1, 3 and 8			Laboratories 1 and 6		
Study sample	GM^a^	GCV (%)^b^	*N*	GM	GCV (%)	*n*	GM	GCV (%)	*n*
10/126	118	17	7	100	7	3	99	17	2
G	56	26	7	47	<1	3	37	18	2
H^c^	111	8	6	101	3	2	92	12	2
I	28	22	7	28	22	3	25	14	2
J	69	22	7	70	<1	3	63	15	2
K	15	48	4	12	23	2	10	16	2
L	318	40	7	361	8	3	335	13	2
M	16	14	7	15	18	3	13	21	2

a Geometric Mean.

b Inter-laboratory Geometric Coefficient of Variation.

c Coded duplicate of IS 16/138.

It was noted that samples G, I and J were tested in the previous study to establish IS 16/138 [19]; and that the GM potency estimates obtained by the VaccZyme ELISA in that study (38, 25 and 62 IU mL^−1^ for samples G, I and J, respectively; *n* = 6) show excellent agreement with the geometric mean of values obtained by laboratories 1 and 6 in this study (37, 25 and 63 IU mL^−1^ for samples G, I and J, respectively; *n* = 2; Table 8). In the same study, geometric mean potencies were obtained by the Vi-PLL ELISA for these samples (43, 27 and 67 IU mL^−1^ for samples G, I and J, respectively; *n* = 2), showing good agreement with the optimum GM values obtained in this study (47, 28 and 70 IU mL^−1^ for samples G, I and J, respectively; *n* = 3; Table 8).

### 3.3 Biotinylated Vi ELISA

The biotinylated Vi ELISA performed by laboratory 6 gave valid estimates of potency relative to IS 16/138 for all samples (Table 4, Table 6), with low levels of intra-laboratory variation (GCV≤8%; Table 5). However, the ranking of samples G, I and J based on the geometric mean potencies differed from that obtained by the Vi-PLL and VaccZyme ELISAs (Table 6 and Fig. 3). Indeed, potency estimates obtained in the previous study for these study samples, then coded as B, D and E, by the same biotinylated Vi ELISA were ranked similarly and did not agree with values obtained by the VaccZyme ELISA [19]. The results of the current study again confirm a low level of concordance between the biotinylated Vi ELISA and the VaccZyme or Vi-PLL ELISAs [19].

## 4 Discussion

The aim of the current study was to evaluate the performance and reproducibility of a standardised Vi-PLL ELISA alongside the commercial VaccZyme ELISA by multiple laboratories. Only participant 6 used their in-house ELISA to test study samples, and thus the emphasis of the current study remains on the comparison of the performance of the Vi-PLL ELISA and the VaccZyme ELISA. The Vi-PLL ELISA has several advantages over the VaccZyme ELISA, in that its procedure is published in the public domain [24,26,27], it uses publicly available biologicals of standardised quality, its availability is not limited to a single source and it is less costly to run. Disadvantages are the lower level of standardization of reagents and the absence of assay specific run controls.

Two participants of the previous study, represented here by laboratory codes 1 and 8, submitted a superior dataset from the Vi-PLL ELISA, which was their in-house ELISA at the time of the previous study [19]. Both participants produced results with low intra-laboratory variability and consistently reported high numbers of valid estimates (see Table 3, Table 4). Indeed, a post-study questionnaire (results not shown) confirmed that participants adhered closely to the Vi-PLL ELISA procedure, and that familiarity with the Vi-PLL ELISA was the most important factor for achieving low intra-laboratory GCVs. A similar observation can be made for participant 6, a national reference laboratory, which performed their in-house Vi-biotinylated ELISA to a higher standard than the Vi-PLL ELISA, which was introduced in this laboratory as part of the current study. Participants 1, 2, 3, 5, 6, 8, 9 and 10 produced Vi-PLL ELISA data that were acceptable by our criteria. However, relative potency estimates produced by laboratory 2 showed the lowest concordance within this group. The cause of this discrepancy is unclear.

Potency estimates in the Vi-PLL ELISA produced by participants 4 and 7, and by participant 5 in the VaccZyme ELISA were less convincing, with several high GCV values (>40%) and a low number of valid estimates (see Table 3, Table 4). We speculate that in these cases limited experience with the two ELISA procedures may have played a part. We note that by criteria less stringent than used in our study these results may well be acceptable for routine serology.

Exclusion of the relative potency estimates from laboratories 2, 4 and 7 resulted in a high level of concordance between the Vi-PLL and VaccZyme ELISAs and confirmed the status of sample K as an outlier (see Table 8 and Fig. 4a and b). We also observed a good agreement with the relative potencies assigned to coded samples reported in the previous study [19].

Thus, despite most of the participating laboratories not being familiar with the Vi-PLL ELISA, a satisfactory performance was achieved in most cases, with good repeatability and reproducibility observed in the study results across the majority of laboratories. Concordance in the relative potency estimates obtained by the Vi-PLL ELISA and the VaccZyme ELISA was also evident for the panel of samples tested.

We conclude that this study demonstrated that the Vi-PLL ELISA is a credible alternative to the VaccZyme ELISA, provided operators are trained and competent, that criteria for reagent quality, for example the use of high molecular weight PLL, are adhered to, that an appropriate reference standard is used, and that assay validity criteria (system and sample suitability criteria) are applied. These measures should enable an optimal performance of the Vi-PLL ELISA. To minimise intra-laboratory variation, it is recommended that a dose response curve of an in-house reference standard calibrated in IU relative to the 1st WHO IS for anti-Vi IgG (human) is included in each run. With such an approach the Vi-PLL ELISA should aid the harmonisation of clinical trial studies with different TCV formulations and allow comparison of serology data from different target groups.
